# Preparation and Characterization of Biomimetic Functional Scaffold with Gradient Structure for Osteochondral Defect Repair

**DOI:** 10.3390/bioengineering10020213

**Published:** 2023-02-06

**Authors:** Li Chen, Li Wei, Xudong Su, Leilei Qin, Zhenghao Xu, Xiao Huang, Hong Chen, Ning Hu

**Affiliations:** Department of Orthopedics, The First Affiliated Hospital of Chongqing Medical University, Chongqing 400016, China

**Keywords:** gradient scaffold, mesenchymal stem cells, osteochondral defect, tissue engineering, regeneration

## Abstract

Osteochondral (OC) defects cannot adequately repair themselves due to their sophisticated layered structure and lack of blood supply in cartilage. Although therapeutic interventions are reaching an advanced stage, current clinical therapies to repair defects are in their infancy. Among the possible therapies, OC tissue engineering has shown considerable promise, and multiple approaches utilizing scaffolds, cells, and bioactive factors have been pursued. The most recent trend in OC tissue engineering has been to design gradient scaffolds using different materials and construction strategies (such as bi-layered, multi-layered, and continuous gradient structures) to mimic the physiological and mechanical properties of OC tissues while further enabling OC repair. This review focuses specifically on design and construction strategies for gradient scaffolds and their role in the successful engineering of OC tissues. The current dilemmas in the field of OC defect repair and the efforts of tissue engineering to address these challenges were reviewed. In addition, the advantages and limitations of the typical fabrication techniques for gradient scaffolds were discussed, with examples of recent studies summarizing the future prospects for integrated gradient scaffold construction. This updated and enlightening review could provide insights into our current understanding of gradient scaffolds in OC tissue engineering.

## 1. Introduction

Articular cartilage forms a durable covering on the articular surfaces of bones, which plays a vital role in keeping joints in good working order. Articular cartilage injury has become a common joint disease that can occur as a result of either progressive degeneration or trauma [1]. Once damaged, articular cartilage has a limited ability to repair itself due to the lack of blood vessels, nerves, and lymph tissues [2]. Recent studies have suggested that the prevalence of cartilage injury in patients undergoing knee arthroscopy ranges from 61% to 63% [3]. Generally, articular cartilage injuries may spread deep into subchondral bones, in which case they are known as osteochondral (OC) defects, which can further accelerate the progression of osteoarthritis and cause a severe socioeconomic burden to society [4]. Unfortunately, current conservative treatments for osteoarthritis are commonly palliative and cannot prevent further joint degeneration [5]. Invasive strategies, including microfracture, autologous chondrocyte implantation (ACI), and arthroplasty, are also applied to treat OC defects, but they are associated with some risks, such as infection, worn-out implants, shortage of donor tissues, and immunogenic responses [6].

With the developments of materials and biomedicine, scaffold-based tissue engineering has become a promising approach to repair OC defects. Indeed, traditional single-phase scaffolds can provide mechanical support for chondrogenic differentiation of stem cells and accelerate cartilage formation [7,8]. However, it is difficult to create high-quality OC tissue that has good integration with surrounding existing tissues [9]. Furthermore, the native OC interface tissue has a distinct structure in terms of cell species and matrix components, which leads to a gradient variation in fiber arrangement, mechanical properties, and function [10]. Therefore, gradient scaffolds mimicking the hierarchical nature of OC tissue are superior to single-phase scaffolds for OC regeneration. To date, a plethora of studies have been conducted to investigate the preparation process and application of gradient scaffolds aiming to regenerate both articular cartilage and the underlying subchondral bone, with the hope of developing better treatment options for OC defects.

This review aims to provide a comprehensive overview of gradient scaffolds in OC tissue engineering, including the main challenges, design concepts, and construction strategies. Specifically, the first section of the paper reviews the physiological properties of OC tissue, followed by a consideration of the difficulties of tissue regeneration and the limitations of conventional therapeutic approaches. Then, we describe in detail OC gradient scaffold tissue engineering strategies, such as the selection of seed cells, bioactive factors, and materials. A later section places particular emphasis on architectural strategies for gradient scaffolds in OC tissue engineering. Moreover, we extensively review the techniques for the fabrication of gradient scaffolds and highlight the advantages and limitations. Finally, the current challenges and future prospects of gradient scaffold-based OC tissue engineering are explored.

## 2. Biology of OC Tissue

**Figure 1:**
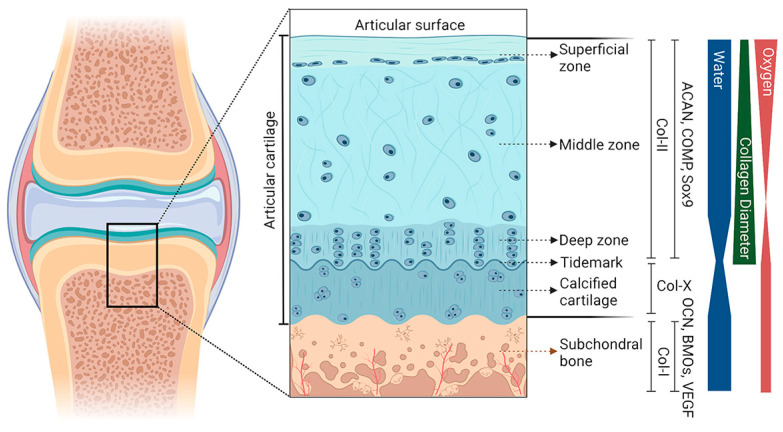
Gradient schematic cross-sectional representation of the OC unit. The different regions (superficial zone, middle zone, deep zone, calcified cartilage, and subchondral bone) and the gradient of the unit are presented. These include collagen type II and X (Col), aggrecan (ACAN), cartilage oligomeric matrix protein (COMP), and Sox9 for articular cartilage; and Col-I, osteocalcin (OCN), bone morphogenetic protein (BMP), and vascular endothelial growth factor (VEGF) for subchondral bone. The thickness of the bands on the right side represents the relative content of each component at the corresponding level.

OC tissue is predominantly composed of two components, hyaline cartilage and subchondral bone (Figure 1). The cartilage layer can be divided into noncalcified and calcified cartilage, and the “tidemark” is used as the boundary. The noncalcified cartilage can be further categorized as three layers starting from the surface, including the superficial zone, the middle zone, and the deep zone. There is a discrete zonal structure with each layer having its own cell distribution, extracellular matrix (ECM) composition, and orientation of collagen fibrils [11]. Primarily, chondrocytes are the major cell type and account for 5–10% of the components in cartilage tissue, which presents different sizes, shapes, and orientations among different zones of the cartilage [12]. The ECM of the cartilage is mainly composed of proteoglycan and collagen, with a small amount of glycoprotein and noncollagenous protein.

The superficial zone, 10–20% of the thickness, contains inert flattened fibroblast-like chondrocytes and a high density of collagen fibrils [13]. These collagen fibrils are considered to be the thinnest (30–35 nm in diameter) and are arranged parallel to the joint surface, which allows the superficial zone to have excellent mechanical properties of shear resistance [14]. From the middle zone to the deep zone, fewer chondrocytes are observed, and the presentation of collagen fibrils switches from random to perpendicular relative to the articular surface. As a transition region of OC tissue, calcified cartilage contains a small number of chondrocytes, and collagen fibrils in this layer are anchored to the subchondral bone. This particular organization is responsible for holding the cartilage and subchondral bone.

The subchondral bone, located beneath the calcified cartilage, is a highly vascularized and biomineralized connective tissue composed of cortical and cancellous bone. Cortical bone can be described as the subchondral bone plate, which is formed of repeated bone units with less porous and limited blood vessels, providing it with a higher compression modulus than that of cartilage. In contrast, cancellous bone relies on bone trabeculae to form a framework and is rich in blood vessels and nerves, thereby generating nutrition for the superficial cartilage [15]. This biphasic structure with the subchondral plate and trabecula enables subchondral bone to absorb the stress load transferred by articular cartilage. Physiologically and mechanistically distinct subchondral bone plate thickness and cancellous bone density vary with regions in the joint [16]. Type I collagen, proteoglycan and its complex, and hydroxyapatite are the primary components of subchondral bone, of which the inner surface is covered with osteoblasts and osteoclasts [17]. Due to the precise arrangement of these structures, the subchondral bone plays a crucial role in shock absorption, mechanical load absorption, and the regulation of metabolism.

## 3. Cells and Bioactive Factors in OC Tissue Engineering

### 3.1. Cells in OC Tissue Engineering

To restore tissues with the same arrangement and function of cell types as those of healthy tissues, certain kinds of cells have been used to initiate the appropriate biological response for OC tissue regeneration. In previous studies, natural cells in joints, mesenchymal stem cells (MSCs), and MSC-derived osteoblasts and chondroblasts have been commonly seeded into the different cartilage and bone layers of the scaffold to regenerate a sufficient ECM for a given tissue. Chondrocytes seem to be an obvious choice for the regeneration of the cartilage compartment since they are capable of simulating the production of new cartilage tissue with the typical characteristics of native hyaline cartilage. Autologous chondrocytes from spare cartilage, on the other hand, are in short supply and tend to dedifferentiate, producing little collagen II, which is characteristic of hyaline cartilage [9,18], and generating collagen I instead [19]. In fact, MSCs are the most common type of cells that are utilized in OC scaffolds [20,21,22]. Their ability to differentiate into chondrocytes and osteoblasts, called pluripotency, enables them to repair different areas of OC tissue.

Among MSCs, bone marrow mesenchymal stem cells (BMSCs) have a high osteochondrogenic potential, but they are limited in number and may also cause pain or morbidity in the donor area [23,24,25]. Adipose-derived mesenchymal stem cells (ADSCs) compensate well for this due to their easy availability [26,27,28]. It is worth mentioning that a study suggested that synovial MSCs (syn-MSCs) have a high capacity for proliferation and chondrogenic differentiation compared to BMSCs, and have thus become a possible option for OC tissue engineering [29]. Mak et al. [30] demonstrated that ADSCs cocultured with articular chondrocytes from osteoarthritis patients could increase the expression of chondrogenic genes, which corroborated the above view. Syn-MSCs also possess chondrogenic potential, retain high transplant survival rates, and undergo rapid proliferation and chondrogenic differentiation compared to BMSCs and ADSCs [31]. Reports have demonstrated that the delivery of syn-MSCs to cartilage defect sites might provide a novel therapeutic modality for the treatment of articular cartilage diseases [32,33,34,35]. Furthermore, another strategy involves a combination of MSCs and tissue-specific cells. Coculturing MSCs and chondrocytes has been shown to promote MSC differentiation into chondrocytes while also preventing chondrocyte phenotypic drift [36,37,38]. Dahlin et al. [39] reported that TGF-β3 induced chondrogenesis in cocultures of chondrocytes and mesenchymal stem cells on biodegradable scaffolds.

Although multiple types of cells have been used to initiate appropriate OC tissue regeneration, there are still some limitations. One question to be considered is how to select appropriate cells for a specific gradient scaffold. The two dominant options include the direct use of already differentiated cells or the use of progenitor cells [40,41]. When differentiated cells are considered, it is recommended that osteoblasts be loaded into a scaffold that mimics the subchondral bone layer, in which a network of inter-connected pores provides space for the growth of blood vessels [42]. For the part of the scaffold designed to regenerate hyaline cartilage, the use of chondrocytes is the natural choice. Since cartilage lacks vascularity, the scaffold structure does not require as large a pore space as the bone layer. The structure of the middle layer of a multi-layer gradient scaffold prevents blood vessels from growing upward into it. When MSCs are used, the strategy of scaffold design differs from the former because it must provide the right cues to guide their differentiation towards the osteogenic and chondrogenic lineage in the appropriate compartments of the structure. It is noted that the use of growth factors in soluble form or bound to the scaffold structure can effectively drive MSCs’ differentiation towards the target lineage [43,44]. In addition, the stimulation of scaffold stiffness also influences the direction of progenitor cell differentiation [45]. Specifically, cells attached to soft materials are more likely to differentiate toward chondrocytes, while cells on stiffer materials will be driven toward the osteogenic lineage. Meanwhile, some studies have pointed out that the extracellular matrix secreted by the pre-inoculated cells triggers an immune response, which favored the application of cell-free scaffolds to promote OC tissue repair [46,47,48]. Overall, with the rapid development of manufacturing technology, novel scaffolds with precisely controlled porosity, density, and morphology are emerging, which are a promising approach to overcome the aforementioned limitations.

### 3.2. Bioactive Factors in OC Tissue Engineering

While the biomaterials that compose the OC scaffold serve as the foundation of the structure, synchronously, bioactive factors play an essential role in promoting the regeneration of articular cartilage and subchondral bone. Growth factors, as well as small drug-like molecules and cytokines, have been shown to direct MSC differentiation into target cells in previous studies.

Growth factors are a class of peptides that regulate tissue development, regeneration, and homeostasis. In particular, members of the transforming growth factor-β (TGF-β) superfamily have received much attention in both bone and cartilage research since they contribute significantly to their development. Typical TGF-βs have been shown to (i) stimulate the proliferation and chondrogenic differentiation of MSCs, (ii) improve ECM production, and (iii) inhibit the degradation of cartilage [49,50,51]. In addition, bone morphogenetic proteins (BMPs), another group of proteins in the TGF-β superfamily, have also been widely studied in the field of cartilage tissue engineering (especially BMP-2, BMP-4, and BMP-7), since they are capable of inducing the synthesis of the ECM and promoting the differentiation of MSCs [52,53,54]. Similarly, insulin-like growth factor (IGF) isoforms (IGF-1 and IGF-2) have been shown to promote the proliferation of chondrocytes and MSCs and induce the synthesis of the ECM [55,56,57]. Other growth factor families, including FGFs and PDGF, are also involved in the construction of OC scaffolds by stimulating the proliferation of chondrocytes and MSCs and maintaining the homeostasis of the cartilage matrix [58,59]. Over the past years, growth factor mimetic peptides representing a unique class of bioactive agents have been derived from the existing growth factors on a large scale and at a low cost [60]. Compared with naturally isolated growth factors, peptides have an advantage in reproducibility, stable efficacy, production efficiency, and higher modifiability [61]. An increasing number of peptides, such as TGF-β and BMP mimetic peptides, ECM-derived peptides, and self-assembling peptides, have shown the ability to promote OC tissue repair [62,63,64,65].

Aside from growth factors, drug-like molecules have also been widely used in OC regeneration tissue engineering due to their easy high-throughput screening and simple, low-cost administration. The sequential addition of small molecules to the scaffold is effective in inducing osteogenesis and chondrogenesis and thus can be used for the treatment of potential OC defects [66,67]. For example, kartogenin, one of the most investigated molecules, has demonstrated cell-homing potential (a molecule capable of attracting local MSCs) and is increasingly applied alone or in combination with other molecules [68]. Dexamethasone, a potent glucocorticoid, has been shown to have anti-metabolic and pro-metabolic effects on cartilage and can be used as an adjunct to OC repair strategies [69]. Other small molecules, including berberine, alendronate (ALN), and 6,8-dimethyl-3-(4-phenyl-1H-imidazol-5-yl) quinolin-2(1H)-one (DIPQUO), have been reported in studies to possess osteogenic properties and are capable of promoting OC regeneration in vivo [20,70,71]. Notably, an important limitation of small molecule drugs is that they are dose-dependent and less target-specific than protein formulations, which may have deleterious effects in certain conditions [72].

Recently, in the context of OC tissue engineering, an increasing number of bioactive factors have been used in combination in scaffolds [73,74]. For instance, Martin et al. [75] reported that a nanofibrous hyaluronic acid scaffold delivering TGF-β3 and stromal cell-derived factor-1α (SDF-1α) improved cartilage regeneration in a large animal model of full-thickness cartilage defects. Specifically, SDF-1α increased the recruitment and infiltration of mesenchymal stem cells (MSCs), and TGF-β3 promoted cartilage tissue formation. This undoubtedly provides a new path for future OC tissue engineering research. However, the release kinetics and bioactivity of scaffolds delivering multiple bioactive factors need more precise and extensive validation.

## 4. Design of Scaffolds in OC Tissue Engineering

### 4.1. Choice of Materials

To restore a tissue to have the same functions as a healthy one, the biodegradation of the scaffold material during an in vivo treatment should closely match the rate of tissue growth in the context of OC tissue engineering. In addition, the material should be constructed from biocompatible materials that do not cause a rejection reaction. Based on these considerations, several categories of materials are used for OC regeneration, including natural biomaterials, synthetic materials, and polymeric scaffolds hybridized with inorganic materials, such as metals and ceramics.

A range of natural biomaterials, including collagen, gelatin, chitosan, alginate, and silk, have been used to fabricate OC scaffolds, benefitting from their resemblance to the extracellular matrix structure and biocompatibility [76,77,78]. Collagen, for example, is a widely used natural polymer since it is a major component of connective tissue. However, the instability and rapid rate of degradation prevent the scaffold from maintaining its structural integrity over time [79]. Gelatin has also been utilized for OC repair due to its facile preparation and good flexibility [80,81]. Because of its insufficient elasticity modulus, gelatin also requires cross-linking with materials, such as hydroxyapatite, bioactive glass, and chitosan, to modify its mechanical strength [82,83,84]. Synthetic materials, such as polyethylene glycol (PEG), polylactic acid (PLA), and polycaprolactone (PCL), are readily available and possess excellent plasticity and mechanical qualities [85,86,87]. Nevertheless, their cytotoxicity, inflammatory reaction, and hydrophobicity restrict their application. Some recent works have revealed that new synthetic polymers, for instance PHAs, possess good characteristics, including nontoxicity, biodegradability, and biocompatibility. However, the application of PHAs is limited due to their weak mechanical and thermal properties, slow degradation rate, lack of bioactivity, and poor hydrophilic properties. Different approaches are needed to overcome these obstacles [88,89].

In general, natural biomaterials have excellent plasticity and biocompatibility and can be easily incorporated into synthetic biopolymers while promoting cell adhesion and proliferation. However, their poor mechanical properties and uncontrollable degradation rates require them to be cross-linked with synthetic materials to enhance scaffold mechanical strength and bio-affinity to host tissues [83]. In addition, there is demand for artificial graft alternatives (scaffolds) with optimized properties in terms of supporting cell adhesion, growth, migration, and differentiation, thereby leading to the formation of new bone and cartilage tissue [90]. By increasing the inventory of suitable doped polymers, composite scaffolds have the potential to become more widely applicable and efficient for different cell and tissue types [91]. For instance, bioactive materials, such as hydroxyapatite, calcium phosphate, and biological ceramics, can encourage biomineralization to restore OC tissue, bringing about a new dimension of gradient scaffold construction [92,93,94].

### 4.2. Architecture of Scaffolds

**Figure 2:**
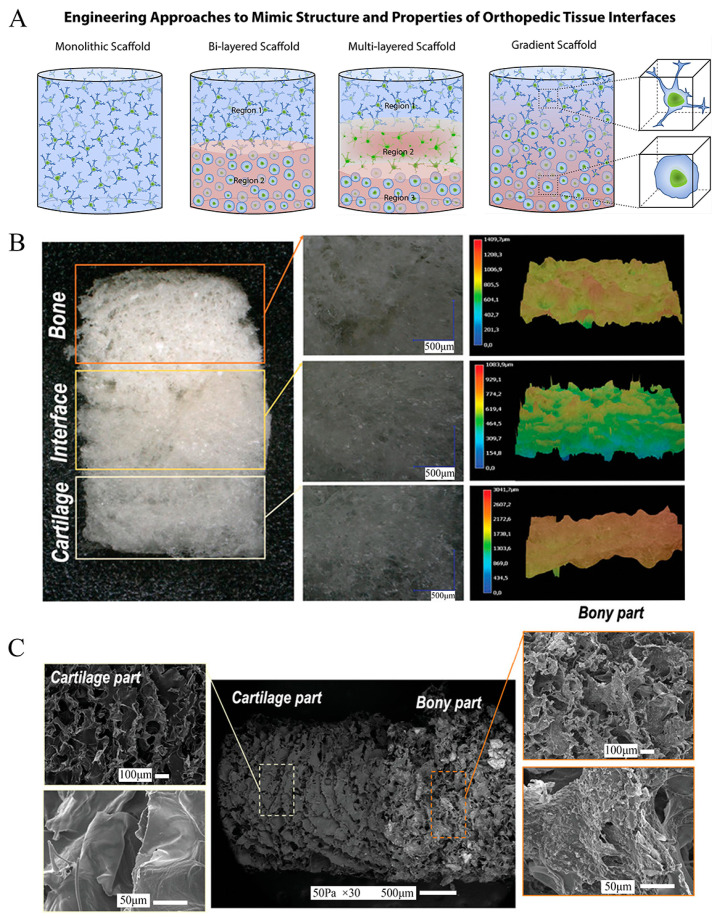
Schematic and representative examples of the design of tissue-engineered osteochondral scaffolds. (**A**) Classification of scaffold strategies based on the number of layers and gradient properties of the designs: Monophasic scaffolds are formed with a single homogeneous layer (left). Discrete gradient constructions include bi-layered (two layers) or multiphase (three or more layers). In the latter, each layer represents a specific region of the OC unit. The continuous gradient scaffolds (right) have a gradual transition between regions which better simulates the original characteristics of the joint. Reproduced with permission from Ref. [95]. Copyright 2016, Elsevier. (**B**) Digital light microscopy images showing the different zones in the multiphase scaffold. Reproduced with permission from Ref. [96]. Copyright 2021, Wiley. (**C**) SEM images illustrating the microstructure and morphological features of the biomimetic scaffold [96].

Material selection and scaffold structural building are interconnected and codependent steps of the OC scaffold design process. To successfully construct a scaffold that conforms to the natural tissue structure of OC tissue, it is necessary to simulate the anatomical and physicochemical properties of cartilage, calcified cartilage, and subchondral bone as closely as possible. Traditional single scaffolds are insufficient to replace the anisotropic OC tissue characteristics, so the concept of gradient scaffolds was introduced. In the last decade, OC scaffolds have evolved from the simplest monolithic scaffolds to bi-layered, multi-layered, and continuous gradient scaffolds (Figure 2). These scaffolds are characterized by different sites with different spatial structures and the bioactive molecules they are loaded with; therefore, they also have different mechanical properties and biochemical environments (Table 1).

#### 4.2.1. Monolithic Scaffolds

Monolithic scaffolds, which are one of the classical techniques for OC repair, are typically referred to as scaffolds that are monophasic and homogeneous in composition and structure. Specifically, monolithic scaffolds merely contain the same amount of one material or a compound of materials that present a spatially homogeneous distribution in their structure and porosity. It has been shown that monolithic scaffolds that rely on different invasive cells and mechanical stimuli can fortify the recruitment and proliferation of chondrocytes as well as osteoblasts [97,98]. Monolithic scaffolds with a single structure, however, tend to simply promote structurally uniform regeneration tissue across the OC defect interface, although they can be produced in several ways to achieve the required disintegration rate, strength, and porosity. Collectively, monolithic scaffolds lack the inherent physical structure and properties required to repair OC tissues, which makes them unable to mimic the biological environment well enough to replace defective OC tissues.

#### 4.2.2. Bi-Layered Scaffolds

To overcome the limitations mentioned above, stratified scaffolds with separate bone and cartilage phases were simultaneously developed [99,100,101]. Bi-layered scaffolds are the most traditional layered scaffolds, which can be used to simulate bone and cartilage tissue separately through the addition of the proper growth factors. Biphasic scaffolds consist of two different materials or two different architectural arrangements with structural variance despite being made up of only one material. Compared with monolithic scaffolds, stratified scaffolds can offer tissues the proper chemical, mechanical, and biological stimulation required for cell division and proliferation. Their hierarchical spatial structure provides an appropriate microenvironment for directing cell/cell and cell/matrix interactions [102,103]. The optimal bi-layered scaffolds are loaded with both chondrogenic and osteogenic growth factors and host cells in a hierarchical fashion. Before their implantation in vivo, a double-chamber bioreactor can be used to culture osteogenic and chondrogenic bone [104]. Bernhardt et al. [105] developed a bi-layered scaffold exclusively from marine collagens supporting both osteogenic and chondrogenic differentiation and found a suitable setup for the in vitro chondrogenic and osteogenic differentiation of BMSCs. A study from Sun et al. [106] demonstrated that a mimetic natural scaffold based on demineralized and decellularized bone and collagen type I (Col-I) allograft showed different stimulation of the osteo- and chondro-responses of cells. In addition, Klimek et al. [107] designed a new curdlan-based scaffold enriched with a protein component, whey protein isolate, as well as a ceramic ingredient, hydroxyapatite granules, via a simple and cost-efficient method. The upper region of the biomaterial was whey protein isolate, which mimicked the smoothness of cartilage. Meanwhile, both phases of the scaffold enhanced cell adhesion, proliferation, and chondrogenic differentiation as well as the osteogenic differentiation of BMSCs and ADSCs in vitro. While numerous related studies have reported acceptable results, a number of bi-layered scaffolds have been approved for OC defect reconstruction, most of which are growth factor free, such as the bilayer MaioRegen®, because its instability poses obstacles for transport and storage [108]. On the other hand, bi-layered scaffolds did not exhibit all of the gradients manifested in OC tissue, which has prompted the development of biomimetic scaffolds with more elaborate construction.

#### 4.2.3. Multi-Layered Scaffolds

As mentioned above, the calcified cartilage layer, an important structure for natural bone–chondral interface connectivity and communication, is a key determinant in maintaining the microenvironment of the two tissues, but it is neglected in the construction of bi-layered scaffolds. The multi-layered discrete OC bionic gradient scaffold remedied the former deficiency by copying the natural structure of cartilage, calcified cartilage, and subchondral bone in OC tissue. The calcified cartilage layer acts as a physical barrier, separating the soft cartilage tissue from the hard subchondral bone (whether physiological or reconstructed), which prevents cartilage ossification due to vascular invasion. Meanwhile, the middle zone provides support for the articular cartilage layer by reducing the mechanical load of conduction and contributes to the integration of the implants with host tissues at the interface [109]. Furthermore, the various depths of the OC layers result in different cellular arrangements, which implies that the distinctive hierarchical structure of OC tissue determines the biological properties of the different layers. Accordingly, to simulate this complex longitudinal structural difference between cartilage and bone, multi-layered scaffolds with more graded physicochemical properties were developed. Materials with different densities, pore sizes, and arrangements were fabricated by suturing, gluing, and press-fitting, helping to achieve a smooth transition between significantly different OC tissues.

Recently, Hejazi et al. [110] reported novel 3D-functionality-graded nanofibrous scaffolds composed of five layers based on different compositions containing polycaprolactone, gelatin, and nanohydroxyapatite for osteo-regeneration and chitosan and polyvinyl alcohol (PVA) for cartilage regeneration. In this design, each layer had a fibrous structure with continuous nanofibers with an improved pore size and porosity of the novel 3D scaffold, and the layer designed for bone regeneration had a satisfying cell proliferation rate. More recently, Liu et al. [23] developed a BMSC-laden multilayer scaffold with methacrylic hyaluronic acid (MeHA)/polycaprolactone, incorporating kartogenin and β-TCP. In addition, MeHA modified with diclofenac sodium-conjugated matrix metalloproteinase-sensitive peptide was induced on the scaffold, which achieved an anti-inflammatory effect. Twelve weeks after its implantation into rabbit knee joints, the grip and ground support force test results illustrated increases in ground support, paw grip force, and walking gait parameters, thus predicting improvements in joint function. However, multi-layered scaffolds demonstrated abrupt and significant changes considering the structural and mechanical properties of the various phases, which frequently led to layer delamination and tissue separation during loading [95].

#### 4.2.4. Continuous Gradient Scaffolds

Continuous gradient scaffolds weaken the concept of layers and are instead constructed as a single matrix preparation with gradient properties, such that the continuous transition gives it greater relevance to most natural systems [111,112,113]. This design not only avoids layer stratification and tissue separation upon loading but also promotes chondrogenic and osteogenic differentiation and ECM deposition of BMSCs. In contrast to discrete gradient scaffolds, continuous OC biomimetic gradient scaffolds have the potential to induce a smooth transition between OC tissue components. In addition, the continuous gradient scaffold formulation reduces the instability of the interface while enabling improved load transfer. Methods of continuous gradient scaffold fabrication have been reported to include buoyancy, magnetic attraction, and electrical attraction techniques in which gradual transitions between separate regions can better simulate the inherent characteristics of the joint [114,115,116].

A number of studies have demonstrated the superiority of continuous gradient scaffolds over monolithic and bi-layered scaffolds in reconstructing OC defects [117,118,119,120]. For example, Radhakrishnan et al. [90] reported a gradient nanoengineered in situ formed by hydrogel with chondroitin sulfate (CS) nanoparticles and nanohydroxyapatite (~30–90 nm) to repair OC defects. Eight-week in vivo experiments on rabbit knees demonstrated that this nanoengineered gradient hydrogel promoted subchondral bone formation and hyaline cartilage regeneration with lateral host tissue fusion while improving mechanical compliance compared to the monolithic group. Recently, Gao et al. [91] created a biohybrid gradient scaffold consisting of a top layer of PACG-GelMA hydrogel-Mn^2+^ and a bottom layer of PACG-GelMA hydrogel–bioactive glass for the repair of OC defects by relying on a 3D printing technique. As shown by in vitro biological experiments, the biohybrid gradient hydrogel scaffold not only promoted cell attachment and spreading but also increased gene expression related to the chondrogenic and osteogenic differentiation of human bone marrow stem cells. Approximately 12 weeks after the in vivo implantation of a rat model, the scaffold significantly promoted the simultaneous regeneration of cartilage and subchondral bone.

Current clinical results with OC scaffolds indicate that multi-layered or continuous gradient tissue-engineered approaches offer the most promising results for patients and their conditions. In terms of biological mechanisms, multilayer scaffolds could mimic the fiber orientation, mechanical strength, pore size, and porosity of the OC unit at each level by stacking layers on top of each other, which induces cell differentiation toward osteogenic and chondrogenic lineages [121]. Meanwhile, the transition layer that mimics calcified cartilage constructs not only acts as a physical barrier to inhibit vascular invasion into cartilage, but also reduces the load from the articular cartilage. However, there is a potential risk of fracture in the multilayer gradient scaffold because of the apparent dispersion between its interfaces [122]. The continuous gradient scaffold compensates well for this due to its smooth transition between layers. It maximally mimics the OC tissue structure for the required physiological loading of the joint, while conferring osteogenic and chondrogenic properties, respectively, through the spatial structural differences between the layers [123,124].

In summary, the field of OC tissue engineering has made remarkable progress in recent years. Diverse novel biomimetic materials have made it easy to integrate scaffolds into surrounding native tissue, while multiphase conceptions have made it possible to reconstruct scaffolds with a similar structure and function to those of natural OC tissue, and continuous gradient scaffolds have reduced the risk of potential fracture of discrete gradient scaffolds. Nevertheless, the creation of more effective and stable OC scaffolds that can reproduce native OC tissue more precisely is necessary for real-world clinical needs. Current clinical results with OC scaffolds suggest that the gradient tissue engineering approach offers the most promising results for patients and their conditions.

**Table 1:** Summary of the definitions and characteristics of the gradient scaffolds by different architecture strategies.

Types	Mimic Structures	Material Composition	Advantages	Limitations	References
Monolithic scaffolds	Articular cartilage or subchondral bone	One material or a compound of materials with a spatially homogeneity of structure and porosity	Easily fabricated Easy quality control, conducive to mass production	Model is overly simplistic Unable to mimic the biological environment	[98,99]
Bi-layered scaffolds	Articular cartilage and subchondral bone	Two different materials, architectural arrangements or bioactive factors	Provided appropriate microenvironment for directing cell/cell and cell/matrix interactions Different bioactive factors impart osteogenic and chondrogenic properties, respectively	Ignores the presence of calcified cartilage layer Risk of potential fracture of discrete gradient scaffolds	[100,101,102,103,104,105,106,107]
Multi-layered scaffolds	Noncalcified cartilage, calcified cartilage, and subchondral bone	Vertical superposition of three or more components	Transition layer acts as a physical barrier to inhibit vascular invasion into cartilage Reducing the load from the articular cartilage	Abrupt transition between OC tissue components Risk of potential fracture of discrete gradient scaffolds	[23,95,109,110]
Continuous gradient scaffolds	Full complexity of the chondro-osseous junction tissue	Continuous change among components with different content, spatial structure, and intensity	Smooth transition between OC tissue components Optimal tissue structure biomimetics	High manufacturing technology requirements Poor reproducibility	[90,91,112,114,115,116,117,123]

## 5. Construction Techniques of Gradient Scaffolds

Sophisticated construct techniques are the basis for gradient scaffold construction. As mentioned above, to achieve the construction of a gradient scaffold with a suitable physiological structure, the choice of manufacturing method is essential in the design process of the OC scaffold, since it enables the achievement of a suitable porosity, pore size, and mechanical strength of the target scaffold. The whole scaffold can be made using a single fabrication method, or individual phases can be made using various techniques. The most widely used techniques for creating OC gradient scaffolds are solvent casting, freeze-drying, electrospinning, microfluidic-based methods, and 3D printing.

### 5.1. Solvent Casting

**Figure 3:**
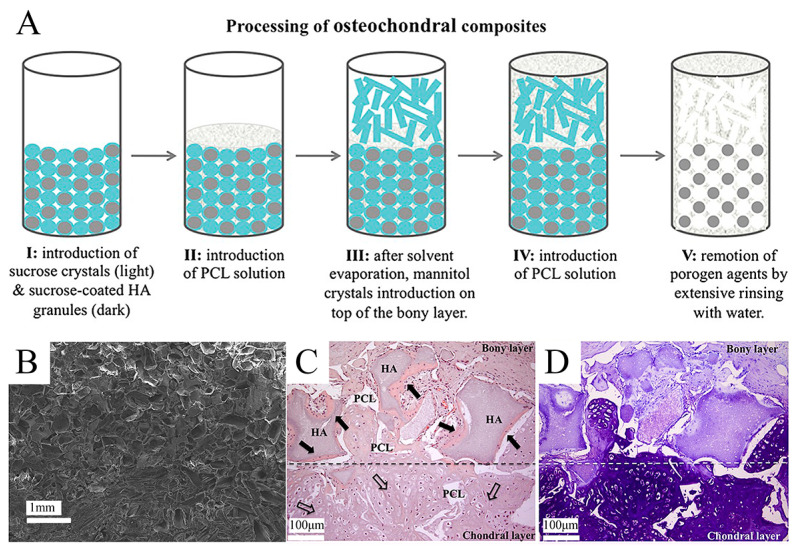
The application of solvent casting/particulate leaching technique for a gradient OC tissue scaffold. Reproduced with permission from Ref. [128]. Copyright 2012, Wiley. (**A**) Schematic model of the structure and process of a highly porous polycaprolactone-based osteochondral composite scaffold produced by the solvent casting/particle leaching technique. (**B**) SEM micrograph of the double-layered scaffold, taken with the secondary electronic detector. (**C**,**D**) H&E and toluidine blue staining of double-layered monolithic osteochondral scaffolds after 9 weeks of implantation in immunodeficient mice (scale bar = 100 µm).

A conventional method for the additive manufacturing of scaffolds, solvent casting, is primarily used to form large-bore scaffolds with randomly oriented holes [125]. It is a relatively straightforward and low-cost technique that is suitable for forming porosity or compositional gradients. In solvent casting, uniformly distributed particles of a certain size, called porogen particles, are dispersed in a polymer solution by size, left to evaporate, and then the matrix is immersed in a solvent to filter out the porogens, resulting in a structure with porous characteristics [126]. This latter step is named particulate leaching. Various types of porogens (e.g., sugar, salt, gelatin, etc.) can be used to produce gradients of pore size while maintaining continuity between the layers of the multi-layered scaffold. This construct can model the porous gradient in bone-forming scaffolds with pore sizes ranging from 50 to 450 μm and porosities between 50% and 90% [127]. In Giannoni et al. [128]’s research study, a highly porous polycaprolactone-based graft material was prepared by solvent casting/particle leaching. The material had a biphasic monolithic structure that mimics OC tissue, avoiding the delamination of two different layers while retaining the cue for selective cartilage regeneration. Pore structure and interconnections were designed to favor in vivo vascularization only at the bony layer (Figure 3). Krok-Borkowicz et al. [129] reported the design and fabrication of an integrated scaffold based on poly(l-lactide-co-glycolide) (PLGA) by a solvent casting/particulate leaching method. Specifically, the scaffold was composed of a PLGA surface modified with collagen type I (PLGA/col-I) or hydroxyapatite (PLGA/HAp) and had pore diameters of 250–300 μm and an 85% total porosity. These highly porous PLGA scaffolds could enhance tissue ingrowth while causing extensive inflammation and inhibiting tissue healing. Lin et al. [130] fabricated a biomimetic integrated biphasic PLGA scaffold with small (200–3300 μm) and large (200–5500 μm) pores by salt leaching. Chondrocytes were loaded in this tyramine-treated biphasic scaffold in its upper and lower regions with different pore sizes to induce differentiation and proliferation, obtaining satisfactory regeneration and integration of OC tissue. Solvent casting and particulate leaching are effective in forming scaffolds with precise pore sizes and complex gradients. However, one of its disadvantages is that the soluble particles cannot be removed from the thicker polymer matrix due to the restricted thickness of the specimens [131].

### 5.2. Freeze-Drying

**Figure 4:**
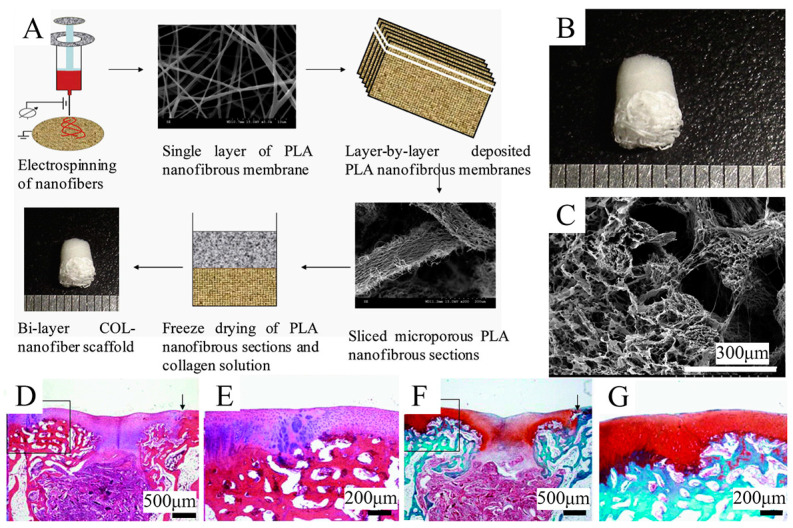
Schematic representation of the combination of freeze-drying and 3D printing techniques used to fabricate the gradient scaffold. Reproduced with permission from Ref. [134]. Copyright 2013, Elsevier. (**A**) Fabrication process of COL-nanofiber scaffolds. (**B**) Macroscopic images of the COL-nanofiber scaffold showing obvious differences between two layers. (**C**) SEM images of the interface between two layers in the bi-layer scaffolds. (**D**,**E**) H&E staining of samples at 6 weeks after surgery. (**F**,**G**) Safranine O staining of samples at 6 weeks after surgery.

Freeze-drying is an effective method that can be used to create several kinds of porous gradient scaffolds [132]. In the freeze-drying technique, the frozen solvent crystals are converted to the gas phase by sublimation, so that the inverse image they leave behind forms the porous scaffolds. This method can yield scaffolds with high interconnectivity, featuring a median pore size of 15–335 μm (with larger pores >200 μm) and porosity of >90%, and have been used at the bone–cartilage interface of various composites. For instance, Zhu et al. [133] engineered stratified porous scaffolds mixed with a chitosan-polycaprolactone (CH-PCL) copolymer and CS through a freeze-drying technique. It has been demonstrated that the porous structures inside collagen/CH-PCL/CS scaffolds possess graded average pore sizes and porosities, showing their potential capability to repair OC defects. Freeze-drying is able to be combined with other techniques, such as electrospinning or 3D printing, to generate functionally graded materials. Zhang et al. [134] combined freeze-drying and electrospinning techniques to fabricate a bilayer collagen/microporous electrospun nanofiber scaffold, which was shown to synergistically promote osteochondral regeneration in vivo (Figure 4). By combining 3D printing and directional freezing, Reed et al. [135] fabricated an acellular, highly porous, hydrophilic chitosan-alginate (Ch-Al) scaffold that substantially improved cell growth and distribution within the scaffold while achieving porous zones that mimicked the zonal structure of articular cartilage. Of note, the application of the freeze-drying method is dependent on cytotoxic solvents; hence, a thorough cleaning is needed to remove solvents and minimize chemical cytotoxicity. Additionally, scaffolds produced by the freeze-drying technique have less homogeneity than those produced by solvent casting.

### 5.3. Electrospinning

Electrospinning capitalizes on the electrostatic repulsion between surface charges, which keeps drawing nanofibers out of a viscoelastic fluid. This technique has been used for a wide variety of natural and synthetic polymers. Pertinently, this method generates fiber architectures that are highly similar to the native ECM, including fibers with tunable diameters and orientations, interconnected porosity, and large surface-to-volume ratios [136]. Meanwhile, the resulting fibers can be modified, functionalized, and stacked by surface modification techniques, such as wet chemistry, plasma treatment, and physical or chemical functionalization with biological ligands or drugs for a controlled delivery [137]. This diversity in postmanufacturing adjustments allows for the modulation of cell adhesion, proliferation, and differentiation, which contributes to the creation of fibrous meshes with variable chemical compositions and associated properties. Recently, bidirectional gradient electrospinning and electrospinning combined with other manufacturing techniques have been developed, representing an attractive route through which the stent properties can be adjusted to reproduce the physiological gradient.

**Figure 5:**
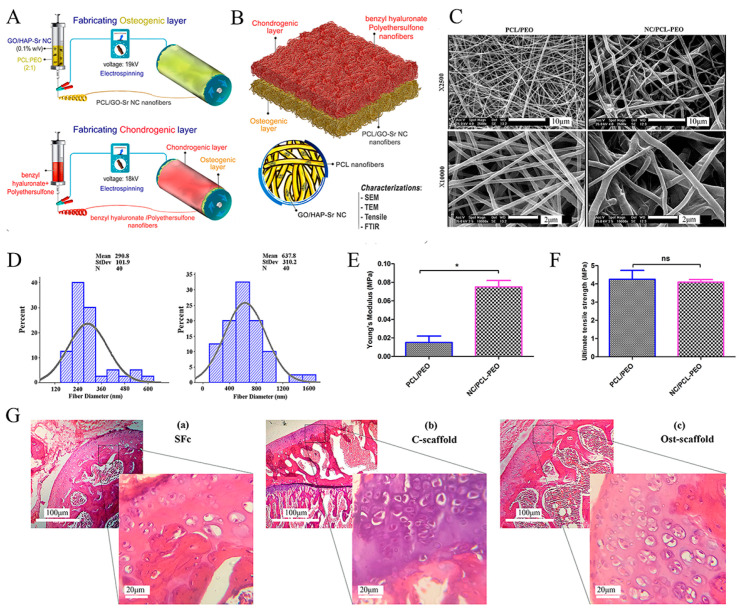
Schematic illustration of a bilayer composite nanofibrous scaffold fabricated by electrostatic spinning technology. Reproduced with permission from Ref. [138]. Copyright 2022, Wiley. (**A**) Electrospinning technique for the formation of osteogenic and chondrogenic layers. (**B**) Incorporating the two layers and characterizing the nanocomposite (NC). (**C**) SEM images of electrospun nanofibers (polycaprolactone polymer (PCL)/PEO and nanocomposite (NC)/PCL-PEO) with two magnifications (×2500 and ×10,000). (**D**) Size distribution of electrospun nanofibers’ diameter, measured and depicted by ImageJ and Minitab v.16 software, respectively. (**E**) Young’s modulus and (**F**) Ultimate tensile stress to assess the mechanical properties of nanofiber scaffolds. * *p* < 0.05, ns: non-significant difference (*p* > 0.05). (**G**) H&E staining of (**a**) scaffold-free control (SFc), (**b**) control scaffold (C-scaffold), and (**c**) osteochondral scaffold (Ost-scaffold). The cells were arranged in columnar orientation, showing the formation of fibrous cartilage in the SFc and C-scaffold. On the other hand, the hypertrophic cells were observed in very limited areas of the Ost-scaffold sample.

Abedin Dargoush et al. [138] prepared an electrospun bilayer nanofibrous scaffold to guide the spatial differentiation of ADSCs (Figure 5). In this process, nanocomposites of hydroxyapatite, strontium, and reduced graphene oxide were combined with polycaprolactone polymers to create the osteogenic differentiation layer. In addition, the chondrogenic differentiation layer was formed with polyethersulfone polymers and benzyl hyaluronan. This electrospun bi-layered scaffold was shown to be biocompatible and to increase the expression of chondrogenic and osteogenic genes, which facilitates the healing of OC tissue. Steele et al. [10] constructed a porous zonal microstructured scaffold from a single biocompatible polymer (poly [ε-caprolactone]) using electrospinning combined with multiple fabrication strategies, including spherical porogen leaching, directional freezing, and melt rewriting. With these approaches, the zonal structure of articular cartilage was simulated, and a stiffness gradient scaffold consistent with the original tissue mechanics was created, resulting in the satisfactory osseointegration and long-term degradation of the microstructured scaffold, which is thought to be of potential benefit in the long-term repair of OC defects.

The main benefit of electrospinning is to fabricate OC gradient scaffolds in a variety of ways that are highly compatible with other manufacturing methods. It enables the mass production of scaffolds with multiple gradients thanks to its advantages of being inexpensive and scalable. However, due to the shear forces generated during extrusion and the involvement of cytotoxic solvents, attention needs to be paid to their adverse effects on cells.

### 5.4. 3D Printing

Three-dimensional (3D) printing, the most representative technology in additive manufacturing, employs computer-aided design (CAD) and layer-by-layer deposition to precisely produce scaffolds with complex structures. The main advantage of 3D printing technology is the precise control of the scaffold architecture, enabling the fabrication of 100% interconnected pore structures and the optimization of the mechanical properties of the scaffold [139]. Several 3D printing methods have been used to create OC tissue scaffolds, mainly including fused deposition modeling (FDM) [140], selective laser sintering (SLS) [141], stereolithography (SLA) [142], digital light processing (DLP) [143], and extrusion-based 3D printing [144].

**Figure 6:**
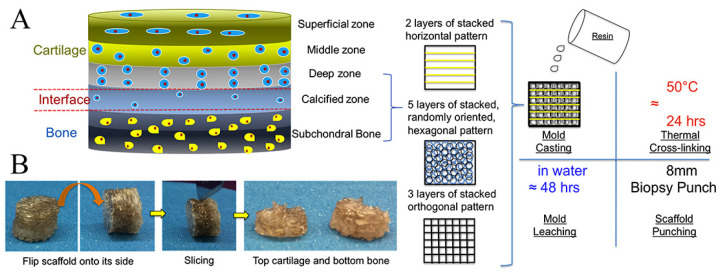
Schematic illustration of multi-layered scaffold and fabrication designed to emulate the structure of OC tissue by 3D printing technique. Reproduced with permission from Ref. [146]. Copyright 2020, Elsevier. (**A**) The superficial region contains horizontally aligned cells and fibers and is represented by horizontal fibers in the FDM mold; the intermediate region containing randomly oriented cells and fibers is represented by a randomly oriented hexagonal pore structure; and the deep region contains vertically aligned cells and fibers represented by orthogonal fibers in the FDM mold. (**B**) Photographic images depicting the resultant scaffold and the sliced bi-layered structure.

**Figure 7:**
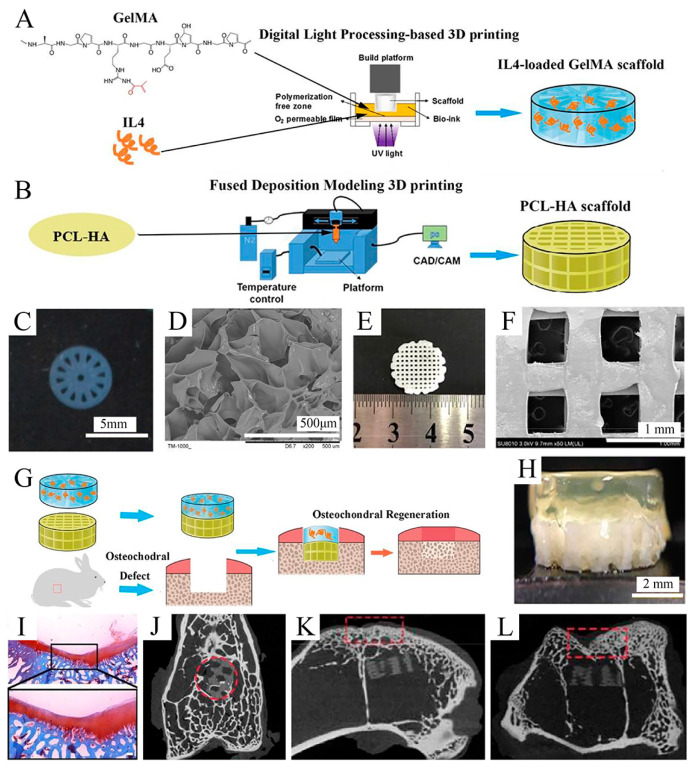
Fabrication and characterization of the upper GelMA layer and the lower PCL-HA layer. Reproduced with permission from Ref. [143]. Copyright 2020, Elsevier. (**A**) Schematic of IL-4-loaded GelMA scaffold prepared by the DLP 3D printing system. (**B**) Schematic of the PCL-HA scaffold prepared by the FDM 3D printing system. (**C**) Macroscopic image of the GelMA scaffold. (**D**) SEM images of the GelMA scaffold. (**E**) Macroscopic image of PCL-HA scaffolds. (**F**) SEM image of PCL-HA scaffolds (×50). (**G**) Schematic of fabricating an IL-4-loaded bi-layered scaffold for rabbit osteochondral regeneration. (**H**) The overall view of the IL-4-loaded bi-layer scaffold. (**I**) Safranine O staining of the repaired cartilage after 16 weeks post-operation. (**J**–**L**) Micro-CT images on the *x* axis (**J**), *y* axis (**K**), and *z* axis (**L**) of the articular joint (*n* = 3 joints) after operation for 16 weeks.

Due to its relatively simple instrumentation, FDM printing has become one of the most popular 3D bioprinting techniques for producing OC tissue engineering scaffolds [145]. Nowicki et al. [146] fabricated gradient OC constructs with different layer geometries by combining fused deposition Model 3D printing with casting techniques. PCL-based shape memory material was used as the OC matrix material, nanocrystalline hydroxyapatite (nHA) was printed onto the subchondral bone layer, and cartilage growth factors were fabricated onto the cartilage layer to achieve spatially appropriate osteogenic and cartilage growth responses (Figure 6). Combining both FDM and DLP techniques, Gong et al. [143] developed a bilayer scaffold that achieved satisfactory repair in a rabbit OC defect model: a radially oriented gelatin methacrylate (GelMA) scaffold printed with DLP simulated the cartilage layer, and a porous PCL and hydroxyapatite (PCL-HA) scaffold printed with fused deposition modeling (FDM) simulated the subchondral layer (Figure 7).

Rather than being nozzle-based, SLA and DLP technology places liquid material in a resin bath into which the build plate is placed with a light source, tracking the programmed pattern and crosslinking only the relevant design. The process continues with layer after layer of material deposition until the object is complete. Castro et al. [142] used table-top stereolithography 3D printing to create a porous, highly interconnected OC scaffold with nHA gradients in a highly porous subchondral bone layer and chondrogenic TGF-1 nanospheres in the cartilage layer for enhanced OC regeneration. This study demonstrated the effectiveness of nanoinks and current 3D printing technologies in the efficient fabrication of OC scaffolds. SLA can typically print feature sizes of 50 microns but is not widely used due to the high upfront and ongoing costs of the system and the limitations of readily available biomaterials [147,148,149].

In addition to the methods mentioned above, new 3D printing techniques are constantly emerging, including cryogenic 3D printing [150], powder-based printing [118], indirect printing [151], phase separation [152] printing, and custom-built printers [153]. These bioprinting technologies facilitate the rapid, on-demand prototyping of OC tissues that possess complex architectural and chemical cues, which enables the formation of increasingly complex gradients, with the ability to integrate multiple materials through the use of multiple print heads [154]. Recent technological advances have propelled 3D printing technology further toward precision medicine in bone and cartilage therapy with the building of in situ gradient scaffolds to repair OC defects [155,156]. Nevertheless, there are still many problems with these new technologies, which include the time-consuming and costly layer-by-layer processing generally required to 3D print at a high resolution, which currently limits their industrial implementation and mass production [157]. The lack of biocompatibility and cell induction in most of the materials used for 3D printing is also notable [158]. Despite these difficulties, OC tissue engineering through 3D printing remains one of the most promising methods available today, offering prospects for the creation of bionic gradient scaffolds.

## 6. Perspectives and Conclusions

The field of tissue-engineered OC scaffolds has grown considerably over the past decade. This review summarizes the biological and mechanical gradients that characterize OC tissue from the superficial cartilage zone to the subchondral bone. We review the current dilemmas in the field of OC defect repair and the efforts of tissue engineering to address these challenges. Although most studies that have constructed gradient scaffolds have shown favorable results for OC tissue regeneration, longer-term clinical studies have not provided satisfactory results; thus, further research on tissue scaffolds for OC regeneration is still needed. The development and combination of cells, novel small-molecule drugs, and various gradient synthesis materials have provided diverse options for gradient scaffold construction. Unfortunately, the lack of homogeneity validation methods makes it difficult to assess their effectiveness and translate these results into the clinic. Based on numerous previous laboratory results, the continuous gradient scaffold is considered to be more promising than the discrete gradient scaffold because it better mimics the native tissue structure without abrupt changes between layers. In terms of manufacturing methods, we have further detailed the most common approaches for the construction of tissue-engineered OC gradient scaffolds. High-resolution 3D printing based on various stacking methods, combined with traditional methods, such as electrostatic spinning, may be a promising way forward. In parallel, new synthetic or composite materials with nontoxic, biodegradable, chondrogenic, and osteogenic properties, which can be used in the above manufacturing technique, are needed to produce OC tissue. To achieve tangible, clinically translatable results, sustained collaboration between all areas of the tissue engineering field is necessary, with a focus on the integration of scaffolds, manufacturing techniques, and various physicochemical cues.

## Data Availability

Not applicable.
